# Clinical and genetic differences between pustular psoriasis subtypes

**DOI:** 10.1016/j.jaci.2018.06.038

**Published:** 2019-03

**Authors:** Sophie Twelves, Alshimaa Mostafa, Nick Dand, Elias Burri, Katalin Farkas, Rosemary Wilson, Hywel L. Cooper, Alan D. Irvine, Hazel H. Oon, Külli Kingo, Sulev Köks, Ulrich Mrowietz, Luis Puig, Nick Reynolds, Eugene Sern-Ting Tan, Adrian Tanew, Kaspar Torz, Hannes Trattner, Mark Valentine, Shyamal Wahie, Richard B. Warren, Andrew Wright, Zsuzsa Bata-Csörgő, Marta Szell, Christopher E.M. Griffiths, A. David Burden, Siew-Eng Choon, Catherine H. Smith, Jonathan N. Barker, Alexander A. Navarini, Francesca Capon

**Affiliations:** a Department of Medical and Molecular Genetics, School of Basic and Medical Biosciences, King's College London, London, United Kingdom; b Department of Dermatology, University Hospital Zurich, Zurich, Switzerland; c Department of Dermatology, Beni Suef University, Beni Suef, Egypt; d Department of Medical Genetics, University of Szeged, Szeged, Hungary; e St John's Institute of Dermatology, School of Basic and Medical Biosciences, King's College London, London, United Kingdom; f Portsmouth Dermatology Unit, Portsmouth Hospitals Trust, Portsmouth, United Kingdom; g Paediatric Dermatology, Our Lady's Children's Hospital Crumlin, and Clinical Medicine, Trinity College Dublin, Dublin, Ireland; h Department of Dermatology, National Skin Centre, Singapore; i Department of Dermatology, University of Tartu, and the Clinic of Dermatology, Tartu University Hospital, Tartu, Estonia; j Department of Pathophysiology, University of Tartu, Tartu, Estonia; k Psoriasis Center at the Department of Dermatology, University Medical Center, Schleswig-Holstein, Campus Kiel, Kiel, Germany; l Department of Dermatology, Hospital de la Santa Creu i Sant Pau, Barcelona, Spain; m Institute of Cellular Medicine, Medical School, Newcastle University and the Department of Dermatology, Royal Victoria Infirmary, Newcastle Hospitals NHS Foundation Trust, Newcastle upon Tyne, United Kingdom; n Department of Dermatology, Medical University of Vienna, Vienna, Austria; o Division of Dermatology, University of Washington School of Medicine, Seattle, Wash; p University Hospital of North Durham and Darlington Memorial Hospital, Darlington, United Kingdom; q Dermatology Centre, Salford Royal Hospital, University of Manchester and the Academic Health Science Centre, Manchester, United Kingdom; r St Lukes Hospital, Bradford, and the Centre for Skin Science, University of Bradford, Bradford, United Kingdom; s MTA-SZTE Dermatological Research Group, Szeged, and the Department of Dermatology and Allergology, University of Szeged, Szeged, Hungary; t MTA-SZTE Dermatological Research Group, Szeged, and the Department of Medical Genetics, University of Szeged, Szeged, Hungary; u Institute of Infection, Inflammation and Immunity, University of Glasgow, Glasgow, United Kingdom; v Department of Dermatology, Hospital Sultanah Aminah, Jeffrey Cheah School of Medicine and Health Sciences, Monash University Malaysia, Johor Bahru, Selangor, Malaysia

**Keywords:** Generalized pustular psoriasis, palmoplantar pustulosis, acrodermatitis continua of Hallopeau, *IL36RN*, *AP1S3*, genotype-phenotype correlation, ACH, Acrodermatitis continua of Hallopeau, ERASPEN, European Rare and Severe Psoriasis Expert Network, GPP, Generalized pustular psoriasis, PPP, Palmoplantar pustulosis, PV, Psoriasis vulgaris

## Abstract

**Background:**

The term pustular psoriasis indicates a group of severe skin disorders characterized by eruptions of neutrophil-filled pustules. The disease, which often manifests with concurrent psoriasis vulgaris, can have an acute systemic (generalized pustular psoriasis [GPP]) or chronic localized (palmoplantar pustulosis [PPP] and acrodermatitis continua of Hallopeau [ACH]) presentation. Although mutations have been uncovered in *IL36RN* and *AP1S3*, the rarity of the disease has hindered the study of genotype-phenotype correlations.

**Objective:**

We sought to characterize the clinical and genetic features of pustular psoriasis through the analysis of an extended patient cohort.

**Methods:**

We ascertained a data set of unprecedented size, including 863 unrelated patients (251 with GPP, 560 with PPP, 28 with ACH, and 24 with multiple diagnoses). We undertook mutation screening in 473 cases.

**Results:**

Psoriasis vulgaris concurrence was lowest in PPP (15.8% vs 54.4% in GPP and 46.2% in ACH, *P* < .0005 for both), whereas the mean age of onset was earliest in GPP (31.0 vs 43.7 years in PPP and 51.8 years in ACH, *P* < .0001 for both). The percentage of female patients was greater in PPP (77.0%) than in GPP (62.5%; *P* = 5.8 × 10^−5^). The same applied to the prevalence of smokers (79.8% vs 28.3%, *P* < 10^−15^). Although *AP1S3* alleles had similar frequency (0.03-0.05) across disease subtypes, *IL36RN* mutations were less common in patients with PPP (0.03) than in those with GPP (0.19) and ACH (0.16; *P* = 1.9 × 10^−14^ and .002, respectively). Importantly, *IL36RN* disease alleles had a dose-dependent effect on age of onset in all forms of pustular psoriasis (*P* = .003).

**Conclusions:**

The analysis of an unparalleled resource revealed key clinical and genetic differences between patients with PPP and those with GPP.

## Graphical abstract

**Figure:**
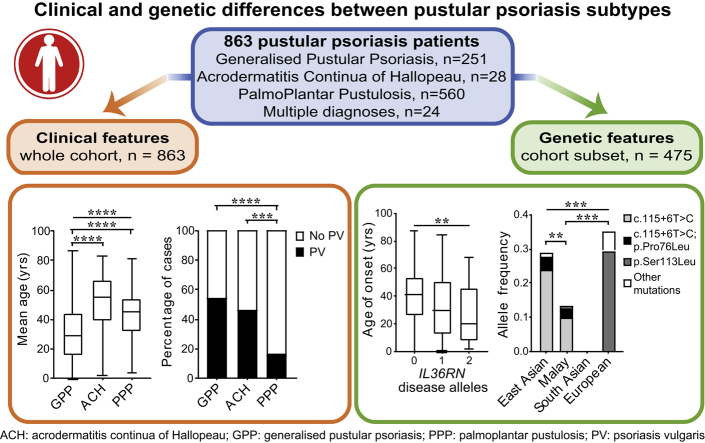

The term pustular psoriasis refers to a group of severe inflammatory skin disorders manifesting with repeated eruptions of painful neutrophil-filled pustules. These conditions can present with acute episodes of skin pustulation and systemic upset (generalized pustular psoriasis [GPP]) or chronic pustular eruptions that affect the palms and soles (palmoplantar pustulosis [PPP]) or the tips of fingers and toes (acrodermatitis continua of Hallopeau [ACH]). Of note, all forms of the disease can be complicated by concurrent psoriasis vulgaris (PV).^1^

We and others have shown that mutations of the gene encoding the IL-36 receptor antagonist *(IL36RN)* are associated with GPP.^2^, ^3^ Although these defects are observed mostly in the homozygous or compound heterozygous state, a number of patients carrying single heterozygous changes have also been reported.^4^

Disease alleles associated with GPP have been identified subsequently in *AP1S3* (encoding a subunit of the adaptor protein 1 complex)^5^ and *CARD14* (encoding a keratinocyte nuclear factor κB adaptor protein).^6^ Of note, *IL36RN*, *CARD14*, and *AP1S3* mutations have also been described in patients with PPP and those with ACH, demonstrating a shared genetic basis for pustular forms of psoriasis.^5^, ^7^, ^8^ Patients harboring disease alleles at 2 distinct loci (*IL36RN* and *AP1S3*; *IL36RN* and *CARD14*) have also been reported.^9^, ^10^ Thus an increasingly complex picture is emerging with evidence of substantial genetic heterogeneity, pleiotropy (the phenomenon whereby a single gene can influence more than 1 trait), and digenic inheritance.

In this context analysis of genotype-phenotype correlations would facilitate stratification of patient cohorts and streamline the genetic diagnosis of disease subtypes. However, rigorous studies have been hindered by the rarity of pustular psoriasis, which has prevented the ascertainment and standardized phenotyping of sizeable patient resources.

Here we sought to address this issue through formation of a multicenter consortium. We brought together 8 tightly phenotyped patient cohorts through a collaboration with the European Rare and Severe Psoriasis Expert Network (ERASPEN). This enabled us to ascertain a unique clinical resource, including 863 unrelated cases and exceeding by nearly 3-fold the size of any published data set. Analysis of this extended cohort revealed very significant differences in the clinical and genetic features of pustular psoriasis subtypes. Specifically, it demonstrated that PPP differs from ACH and GPP in terms of patients’ demographics, disease presentation, and underlying genetic abnormalities.

## Methods

### Patient ascertainment

This research was carried out in accordance with the principles of the Declaration of Helsinki and was approved by the ethics committees of participating institutions. Written informed consent was also obtained from all participants. The study aligned 8 patient cohorts (n = 863) recruited in the reference centers listed in Table E1 in this article's Online Repository at www.jacionline.org. The largest resource (n = 255 British and Irish cases) was provided by St John's Institute of Dermatology (London, United Kingdom) and combined a historical data set (n = 177) with patients ascertained prospectively (n = 78) through the Anakinra in Pustular Psoriasis, Response in a Controlled Trial (APRICOT) clinical trial (EudraCT no. 2015-003600-23) and its sister mechanistic study, Pustular Psoriasis, Elucidating Underlying Mechanisms (PLUM). An additional 40 affected subjects (listed as “others” in Table E1) were recruited outside the main reference centers by clinicians who sent individual samples to the ERASPEN Consortium or St John's Institute of Dermatology.

Pustular psoriasis was diagnosed by expert dermatologists based on direct clinical examination, with the ERASPEN consensus criteria^1^ used in at least 506 cases. The observation of primary, sterile, macroscopically visible pustules affecting nonacral skin (GPP), palms/soles (PPP), or the nail apparatus (ACH) was the main inclusion criterion. Conversely, the occurrence of pustules restricted to the edges of psoriatic plaques represented an exclusion criterion.

### Mutation screening

*IL36RN*, *AP1S3*, and *CARD14* mutations were screened by using Sanger sequencing in 473 patients for whom DNA was available. Primer sequences and cycling conditions have been described elsewhere.^3^, ^5^, ^6^ Nucleotide substitutions were identified by using Sequencher 4.9 (Gene Codes, Ann Arbor, Mich). The deleterious effect of the newly identified c.115+5G>A mutation was confirmed by using Spliceman and MaxEntScan,^11^, ^12^ whereas the pathogenic potential of *CARD14* alleles was assessed with Combined Annotation Dependent Depletion (CADD).^13^

### Statistics

The clinical and demographic characteristics of study participants were analyzed by using a binomial test (to establish the presence of a sex bias among patients with pustular psoriasis), the χ^2^ test with the Yates correction (to analyze differences in the prevalence of PV and proportion of affected female subjects across disease types), and a Kruskal-Wallis test followed by the Dunn multiple comparison test (to analyze differences in age of onset between PPP, ACH, and GPP cases). Analysis of genetic data was based on a χ^2^ test with the Yates correction (to compare the frequency of disease alleles in PPP, ACH, and GPP cases and the combined prevalence of *IL36RN* mutations across ethnic groups) and a 1-tailed Fisher exact test (for association between the *IL36RN* p.Ser113Leu allele and PPP). Genotype-phenotype correlations were investigated by implementing logistic (for PV concurrence and sex ratios) and linear (for age of onset) regression analysis with disease subtype as a covariate. All tests were implemented in R software.^14^

Patients with multiple diagnoses were excluded from all statistical analyses because they could not be assigned to a single disease group.

## Results

### Age of onset and PV concurrence rates vary significantly among disease subtypes

As members of the ERASPEN network, we previously defined consensus criteria for the diagnosis of pustular psoriasis.^1^ Here we build on this work to describe the presentation of key disease features, as observed in clinical practice. We analyzed 863 unrelated patients, the majority of whom (823/863 [95.4%]) were recruited through 6 European, 1 North African, and 1 Asian reference center (Table I and see Table E1). Of note, key patients' demographics (male/female ratios and mean age of onset for various disease types) were comparable across these cohorts (see Table E2 in this article's Online Repository at www.jacionline.org).

**Table I:** Summary description of the patient cohort

	Ethnicity				Sex			Clinical diagnosis						Total
	European	Asian	African	Other∗	Female	Male	Unknown	ACH	PPP	GPP	ACH + GPP	ACH + PPP	GPP + PPP	
Total	591	161	78	33	620	233	10	28	560	251	9	4	11	863

∗ Includes unknown ethnicity (n = 19), mixed ethnicity (n = 4), and Middle Eastern (n = 4), Finnish (n = 2), Filipino (n = 1), Hispanic (n = 1), Jamaican (n = 1), and Romani (n = 1) ethnicity.

While patients with GPP (251/863 [29.1%]) and PPP (560/863 [64.9%]) accounted for most of the data set, the ACH sample was substantially smaller (28/863 [3.2%]), reflecting the extreme rarity of this condition. Of note, the concurrence of multiple disease forms (most notably GPP with ACH and GPP with PPP) was reported in a small percentage of affected patients (24/863 [2.8%]).

A number of comorbidities were observed, with diabetes and hypertension figuring most prominently, regardless of the patient's ethnicity (see Table E3 in this article's Online Repository at www.jacionline.org). In keeping with published associations,^15^ we also found that 11 (3.9%) of 281 European patients with PPP had autoimmune thyroid disease.

**Fig 1:**
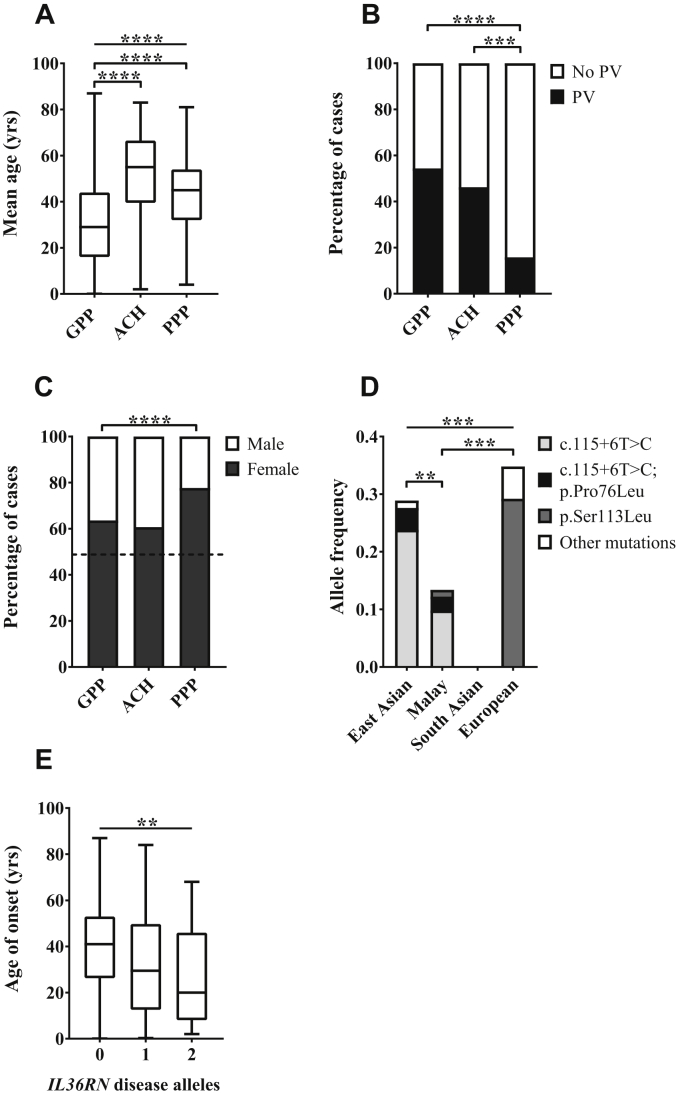
Features of pustular psoriasis observed in the disease cohort. **A,** Mean age of onset was compared across disease groups by using a Kruskal-Wallis test followed by the Dunn multiple comparison test. **B,** Differences in PV concurrence were analyzed with a χ^2^ test. **C,** Differences in the proportion of affected female subjects were assessed by using a χ^2^ test. The *dashed line* indicates the percentage of female subjects in the general population. **D,** Differences in combined frequency of *IL36RN* mutations were assessed across ethnic groups by using a χ^2^ test. Pairwise comparisons were undertaken with the Fisher exact test. The analysis was restricted to patients with GPP because this is the only group for which data were available for multiple ethnicities. *Other mutations* indicates alleles seen only once in the cohort. The notation c.115+6T>C; p.Pro76Leu refers to patients carrying the 2 variants on the same haplotype. **E,** Effects of *IL36RN* mutations on age of onset were assessed by using linear regression. **P < .01, ***P < .001, and ****P < .0001.

Mean age of onset differed considerably across disease types and was lower in patients with GPP (31.0 ± 19.7 years) than in those with PPP (43.7 ± 14.4, *P* = 9.3 × 10^−19^) and those with ACH (51.8 ± 20.4, *P* = 1.2 × 10^−7^; Fig 1, *A*, and see Table E2). Despite these marked differences, there was substantial heterogeneity within the individual disease cohorts, with very early-onset (<10 years) and very late-onset (>70 years) cases observed in all forms of pustular psoriasis.

Although the prevalence of PV in the overall data set (29.1%) was much greater than that reported for the general population (2% to 3%), concurrence rates varied among disease variants. In particular, the frequency of PV among patients affected by PPP (15.8%) was significantly lower than that seen in the ACH (46.2%, *P* = .0004) and GPP (54.4%, *P* = 2.2 × 10^−16^) groups (Fig 1, *B*). Although the latter result was driven in part by a very high prevalence of PV among Malaysian patients with GPP (see Table E2), the difference remained significant (*P* = .01) when the sizeable Malaysian cohort (n = 138) was removed from the analysis. Thus our investigations have demonstrated key differences between disease subtypes, highlighting PPP as a late-onset condition with low PV concurrence.

### PPP is the form of pustular psoriasis most influenced by sex and smoking status

It has been reported that female patients and smokers are at greater risk of PPP than male patients and nonsmokers.^16^ Here we observed a degree of sex bias in all forms of pustular psoriasis as the female/male ratio was 1.5 in patients with ACH, 1.7 in patients with GPP, and 3.5 in patients with PPP. The distortion in sex ratios observed in GPP and PPP was statistically significant (*P* < 10^−5^ and *P* < 10^−15^, respectively) and readily recognizable in individual cohorts (see Table E2). Of note, the difference between the PPP and GPP female/male ratios was also significant (*P* = 5.8 × 10^−5^), highlighting PPP as the condition most influenced by sex-related factors (Fig 1, *C*).

In our data set 79.8% (249/312) of patients with PPP for whom data were available were current or past smokers. Of interest, the rate of PV concurrence was much greater in patients with PPP who smoked (or had done so in the past) compared with those who did not (12.4% vs 1.6%, *P* = .009), suggesting that cigarette smoking can modulate disease manifestations. In fact, smoking has a well-documented effect on aryl hydrocarbon receptor signaling,^17^ a pathway that modulates the severity of inflammation in psoriatic skin.^18^

Although the ACH sample was too small for analysis, the percentage of smokers in the GPP data set (26/96 [28.3%]) was significantly less than that observed in patients with PPP (*P* < 10^−15^), indicating that the adverse effect of cigarette smoking is specific to the latter condition.

### Definition of a patient subset for genetic analysis

Having investigated the key clinical manifestations of pustular psoriasis, we sought to define their relationship with the patient's genotype. For this purpose, we examined the mutation status of 473 affected subjects for whom DNA was available (see Table E1). We collated genetic data previously generated by our group (n = 358)^4^, ^5^, ^6^, ^9^ while also examining 115 newly recruited cases. Importantly, Table E4 in this article's Online Repository at www.jacionline.org shows that the patient subset screened for mutations is representative of the broader data set, suggesting that the findings obtained in this sample can be generalized to the whole resource.

### Frequency of *IL36RN* mutations differentiates PPP from ACH and GPP

The *IL36RN* coding sequence and exon/intron junctions were screened in the entire patient resource, uncovering 66 patients (4 with ACH, 45 with GPP, 12 with PPP, and 5 with multiple diagnoses) with disease alleles (Table II and see Table E5 in this article's Online Repository at www.jacionline.org). Thirty-six of these subjects harbored biallelic (homozygous/compound heterozygous) changes, with the remaining 30 carrying monoallelic (single heterozygous) variants. All the observed mutations had been described previously, except for a c.115+5G>A splicing variant uncovered in a North American patient with GPP (see Table E5).

**Table II:** *IL36RN* and *AP1S3* mutation frequencies across disease types

	ACH	GPP	PPP	Multiple diagnoses
No. of *IL36RN*-positive patients∗	4/23 (17.4%)	45/190 (23.7%)	12/234 (5.1%)	5/18 (27.8%)
*IL36RN* mutation count (frequency)	7/46 (0.15)	72/380 (0.19)	15/468 (0.03)	8/36 (0.22)
No. of *AP1S3*-positive patients∗†	2/19 (10.5%)	4/37 (10.8%)	14/212 (6.6%)	4/11 (36.4%)
*AP1S3* mutation count (frequency)	2/38 (0.05)	4/74 (0.05)	14/424 (0.03)	4/22 (0.18)

∗ Patients were classified as “positive” if they were carrying at least 1 mutation at the examined locus.

† p.Phe4Cys and p.Arg33Trp mutations have no frequency in East Asian populations and therefore were not screened in patients from this ethnic group.

*IL36RN* disease alleles were present in a variety of ethnic groups, with the greatest prevalence observed among patients of European (34.7%) and East Asian (28.8%) descent (Fig 1, *D*). Although we did not detect any rare changes in the 21 South Asian cases we examined, a homozygous p.Leu21Pro mutation has been described in a Pakistani GPP pedigree,^19^ suggesting that deleterious *IL36RN* alleles can also be found within the Indian subcontinent.

The proportion of subjects harboring *IL36RN* disease alleles was greater in GPP and ACH (23.7% and 18.2%, respectively) compared to PPP (5.2%). Patients with GPP and those with ACH were also more likely to carry biallelic mutations compared to individuals affected by PPP (see Table E5). As a result, the prevalence of *IL36RN* mutations was significantly increased in patients with GPP (0.19) and ACH (0.16) compared with that in patients with PPP (0.03; *P* = 1.9 × 10^−14^ and .0018, respectively; Table II). Nonetheless, the association between *IL36RN* mutations and PPP, which has been recently questioned,^10^ was statistically significant. In fact, an analysis of the recurrent p.Ser113Leu variant showed that its frequency in British patients was almost 10 times greater than that observed in population-matched control subjects (*P* = 9.3 × 10^−8^; odds ratio, 10.8; 95% CI, 5.3-22.0; Table III).

**Table III:** Association between *IL36RN* p.Ser113Leu and PPP

	p.Ser113Leu	WT
Cases∗	11 (3.6%)	291 (96.4%)
Control subjects†	26 (0.4%)	7402 (99.6%)

*WT*, Wild-type.

∗ British patients only.

† Control subjects from publicly accessible cohorts (TWINSUK and ALSPAC).

We next sought to determine whether *IL36RN* alleles were associated with key features of pustular psoriasis across disease subtypes. Therefore we implemented a regression analysis using clinical diagnosis as a covariate. Although we did not observe a consistent effect of *IL36RN* mutations on PV concurrence (see Table E6 in this article's Online Repository at www.jacionline.org), we found a significant association with early age of onset (*P* = .003; Fig 1, *E*), which was observed in all 3 forms of the disease (see Table E6). Thus *IL36RN* alleles have shared genetic effects across pustular psoriasis subtypes but occur at a very low frequency among patients with PPP.

### *CARD14* mutations are observed in only a small minority of cases

Although a sizeable patient subset (n = 106/473) was sequenced for the entire *CARD14* coding region, a targeted screening of exons 3 and 4 was undertaken in the rest of the sample, given that the only disease alleles associated with pustular (p.Asp176His) or plaque (p.Gly117Ser) psoriasis map to this mutation hotspot.^6^, ^20^, ^21^

We found 3 previously described^6^ GPP patients of Chinese descent bearing the p.Asp176His variant. We did not detect any *CARD14* substitutions among European patients with GPP but observed 5 British patients with PPP harboring rare nonsynonymous changes with deleterious potential (see Table E7 in this article's Online Repository at www.jacionline.org). Although most of the above subjects (6/8 [75%]) had concurrent PV, the small size of the data set prevented us from establishing genotype-phenotype correlations.

### *AP1S3* mutations occur with comparable frequency across disease types

Although a substantial patient subset (n = 249) was screened for the entire coding region, the rest were sequenced only for exon 2, given that the only known *AP1S3* mutations (p.Phe4Cys, p.Arg33Trp) map to this genomic segment 2.^5^, ^9^ This revealed 24 European cases (2 patients with ACH, 4 with GPP, 14 with PPP, and 4 with multiple diagnoses) bearing the p.Phe4Cys or p.Arg33Trp changes (see Table E8 in this article's Online Repository at www.jacionline.org). No additional mutations were observed in the subjects who were screened for the entire coding region. Of note, 3 patients (2 with GPP and 1 with PPP) carried both *AP1S3* and *IL36RN* disease alleles (see Table E9 in this article's Online Repository at www.jacionline.org).

The prevalence of *AP1S3* mutations was not significantly different across disease types (Table II) and did not seem to influence PV concurrence or age of onset (see Table E10 in this article's Online Repository at www.jacionline.org). However, it was noteworthy that almost all patients with *AP1S3* disease alleles (23/24 [95.8%]) were female. Although this observation was not statistically significant (*P* = .06), a trend toward female overrepresentation was apparent in all clinical variants (see Table E10), suggesting that the penetrance of *AP1S3* mutations might be modified by sex-specific factors, such as hormone levels or X-linked modifiers.

## Discussion

The purpose of our study was to robustly define clinical and genetic features of pustular psoriasis by investigating a patient cohort of unprecedented size.

Initially, we sought to define the presentation of the various clinical variants through a rigorous statistical analysis of key phenotypic features. This work, which builds on the definition of consensus diagnostic criteria by ERASPEN,^1^ underscores the importance of collaborative efforts in the analysis of rare diseases. Here a common case report form was used in all prospectively recruited cases, enabling standardized patient phenotyping and robust data collection. The participation of multiple centers also allowed us to monitor the effects of ascertainment bias and show that key patients’ demographics were comparable across the various data sets.

Our analysis demonstrated novel and significant differences between disease subtypes. Specifically, it showed that PPP is associated with patients’ demographics (very high prevalence of female subjects and smokers), clinical (low rates of PV) and genetic features (low prevalence of *IL36RN* mutations) that are clearly distinct from those observed in ACH and GPP. Given that abnormal IL-36 signaling has now been implicated in the pathogenesis of plaque psoriasis,^22^ it is tempting to speculate that these observations might be correlated with each other and that the decreased prevalence of PV in PPP might be linked to the low frequency of deleterious *IL36RN* alleles in this patient group.

We also found that *IL36RN* mutations are associated with an earlier age of onset across all variants of pustular psoriasis. This validates the results we obtained originally in patients with GPP^4^ and indicates that *IL36RN* should be prioritized for mutation screening when patients have disease symptoms before the age of 30 years (40 years in the case of ACH/PPP). Given that biologics that counter the effect of *IL36RN* mutations by blocking IL-36 signaling are now under development,^23^ such targeted screening could have important implications for patient management.

Our study showed that *IL36RN* mutations are the most frequent genetic abnormality observed in pustular psoriasis. In fact, deleterious *AP1S3* alleles were found in only 7% to 10% of patients, and *CARD14* variants were observed in a very small number of affected subjects. Importantly, our analysis demonstrated that known genes account only for a minority of disease cases. This is especially the case in patients with PPP, in whom the combined frequency of *AP1S3* and *IL36RN* mutations is less than 10%. Therefore additional studies will be needed to illuminate the genetic landscape of this condition, facilitate its diagnosis, and better understand the correlation between genotype and clinical phenotype. Although the discovery of novel genetic determinants has thus far been hindered by the rarity and heterogeneous nature of the disease, the ascertainment and rigorous phenotyping of our clinical resource lays a robust foundation for future gene identification studies.

**Clinical implications**

The association between *IL36RN* mutations and early-onset pustular psoriasis defines a patient group that should be prioritized for *IL36RN* screening and might benefit from the development of IL-36 inhibitors.

## Supplementary data

Tables E1-E10
