# Supplementary material for: Clinical and genetic differences between pustular psoriasis subtypes
Source: J Allergy Clin Immunol. 2019 Mar;143(3):1021–6. doi: 10.1016/j.jaci.2018.06.038 (PMC6403101; doi:10.1016/j.jaci.2018.06.038)
Supplement: Tables E1-E10 [file mmc1.docx]

**Table E1.** Breakdown of patient cohorts by ethnicity and diagnosis.

|  |  |  | **Clinical Diagnosis** | | | |  |  |
| --- | --- | --- | --- | --- | --- | --- | --- | --- |
| **Cohort**  **(recruiting centre)** | **Ethnicity** |  | **ACH** | **GPP** | **PPP** | **Multiple diagnoses^1^** | **DNA available** | **Total** |
| UK/Ireland  (St John’s Institute of Dermatology, London) | European |  | 6 | 31 | 169 | 15 |  |  |
|  | Asian |  | - | 3 | 9 | - |  |  |
|  | Other |  | 1 | 2 | 19 | - | 221 | **255** |
| Malaysia  (Hospital Sultanah Aminah, Johor Bahru) | Asian |  | - | 130 | - | 6 |  |  |
|  | Other |  | - | 2 | - | - | 137 | **138** |
| ERASPEN-Austria  (Medical University of Vienna) | European |  | - | - | 118 | - |  |  |
|  | Asian |  | - | - | 1 | - |  |  |
|  | Other |  | - | - | 1 | - | - | **120** |
| ERASPEN-Egypt  (Beni Suef University, Cairo) | African |  | 1 | 51 | 22 | - | - | **74** |
| ERASPEN-Switzerland  (University Hospital of Zurich) | European |  | 14 | 3 | 53 | - | 61 | **70** |
| ERASPEN-Germany  (University Medical Centre Schleswig-Holstein, Kiel) | European |  | 2 | - | 33 | 2 | - | **37** |
| Hungary  (University of Szeged) | European |  | - | - | 25 | - | 25 | **25** |
| ERASPEN-Estonia  (Tartu University Hospital) | European |  | - | - | 103 | - |  |  |
|  | Other |  | - | - | 1 | - | - | **104** |
| Others  (various sources) | European |  | - | 11 | 6 | 1 |  |  |
|  | Asian |  | 1 | 11 | - | - |  |  |
|  | Other |  | 3 | 7 | - | - | 29 | 40 |
| **Total** |  |  | **28** | **251** | **560** | **24** | **473** | **863** |

^1^Multiple diagnoses: patients suffering from multiple forms of the disease, i.e. ACH+GPP (n=9), ACH+PPP (n=4) or GPP+PPP (n=11)

**Table E2.** Phenotype characteristics of the major cohorts^1^

|  |  |  | **Sex** | | |  | **PV status** | | |  | **Age of onset (yrs)** | |
| --- | --- | --- | --- | --- | --- | --- | --- | --- | --- | --- | --- | --- |
| **Cohort** |  |  | **Female** | **Male** | **Unknown** |  | **PV** | **No PV** | **Unknown** |  | **Mean** | **SD** |
| **UK/Ireland** | GPP |  | 27  (75.0) | 9  (25.0%) | - |  | 16  (41.7%) | 17  (44.4%) | 3  (13.9%) |  | 31.8 | 26.3 |
|  | PPP |  | 151  (76.6%) | 44  (22.3%) | 2  (1.0%) |  | 47  (23.9%) | 119  (60.4%) | 31  (15.7%) |  | 42.4 | 15.9 |
| **Malaysia** | GPP |  | 90  (68.2%) | 41  (31.1%) | 1  (0. 7%) |  | 102  (77.3%) | 29  (22.0%) | 1  (0.7%) |  | 33.3 | 17.1 |
| **Austria** | PPP |  | 103  (85.8%) | 17  (14.2%) | - |  | 14  (11.7%) | 106  (88.3%) | - |  | 42.2 | 13.7 |
| **Egypt** | GPP |  | 21  (41.2%) | 30  (58.8%) | - |  | 4  (7.8%) | 47  (92.2%) | - |  | 27.9 | 18.4 |
| **Switzerland** | PPP |  | 39  (73.6%) | 14  (26.4%) | - |  | 14  (26.4%) | 38  (71.7%) | 1  (1.9%) |  | 45.1 | 15.3 |
| **Germany** | PPP |  | 25  (75.7%) | 6  (18.2%) | 2  (6.1%) |  | 3  (9.1%) | 29  (87.9%) | 1  (3.0%) |  | 42.2 | 16.1 |
| **Hungary** | PPP |  | 20  (80%) | 5  (20%) | - |  | 3  (12%) | 22  (88%) | - |  | 49.5 | 12.4 |
| **Estonia** | PPP |  | 83  (79.8%) | 20  (19.2%) | 1  (1.0%) |  | - | 103  (99.0%) | 1  (1.0%) |  | 47.0 | 10.9 |

**^1^**Data only shown for cohorts including ≥25 patients with the same clinical diagnosis

**Table E3.** Frequently observed co-morbidities

|  |  |  |  | **GPP** |  | **PPP** |
| --- | --- | --- | --- | --- | --- | --- |
| **Ethnicity^1^** |  | **Co-morbidity** |  | **N**  **(%)** |  | **N**  **(%)** |
| African |  | Diabetes |  | 8/51  (15.7%) |  | - |
|  |  | Hypertension |  | 6/51  (11.8%) |  | 1/22  (4.6%) |
|  |  | Autoimmune thyroid disease |  | - |  | - |
| European |  | Diabetes |  | 3/23  (13.0%) |  | 28/285  (9.8%) |
|  |  | Hypertension |  | 6/23  (26.1%) |  | 56/285  (19.7%) |
|  |  | Autoimmune thyroid disease |  | - |  | 11/281  (3.9%) |
| Asian^2^ |  | Diabetes |  | 2/16  (12.5%) |  | n/a |
|  |  | Hypertension |  | 2/16  (12.5%) |  | n/a |
|  |  | Autoimmune thyroid disease |  | - |  | n/a |

^1^The Asian dataset includes East Asians, South Asians and Malays. The information relating to Asian PPP patients was not analysed, as the sample was too small (n=10) to derive any meaningful percentages.

**Table E4.** Clinical and demographic features of the patients who were screened for mutations^1^

|  |  |  | **Sex** | | |  | **PV status** | | |  | **Age of onset (yrs)** | |
| --- | --- | --- | --- | --- | --- | --- | --- | --- | --- | --- | --- | --- |
| **Diagnosis** | **Cohort** |  | **Female** | **Male** | **Unknown** |  | **Has PV** | **No PV** | **Unknown** |  | **Mean** | **SD** |
| **GPP** | Screened cases |  | 128  (67.0%) | 59  (30.9%) | 4  (2.1%) |  | 125  (65.5%) | 56  (29.3%) | 10  (5.2%) |  | 31.4 | 19.6 |
|  | Entire dataset |  | 157  (62.5%) | 90  (35.9%) | 4  (1.6%) |  | 131  (52.2%) | 110  (43.8%) | 10  (4.0%) |  | 31 | 19.7 |
| **ACH** | Screened cases |  | 14  (60.9%) | 9  (39.1%) | - |  | 10  (43.5%) | 11  (47.8%) | 2  (8.7%) |  | 55.1 | 19.2 |
|  | Entire dataset |  | 17  (60.7%) | 11  (39.3%) | - |  | 12  (42.9%) | 14  (50.0%) | 2  (7.1%) |  | 51.8 | 20.4 |
| **PPP** | Screened cases |  | 182  (75.5%) | 57  (23.7%) | 2  (0.8%) |  | 57  (23.7%) | 155  (64.3%) | 29  (12.0%) |  | 43.2 | 15.2 |
|  | Entire dataset |  | 431  (77.0%) | 124  (22.1%) | 5  (0.9%) |  | 83  (14.8%) | 443  (79.1%) | 34  (6.1%) |  | 43.7 | 14.4 |

^1^ The features observed in the broader dataset are shown for reference

**Table E5.** Mutations observed in *IL36RN* positive^1^ patients

| **Sample ID**^2^ |  | **Diagnosis** |  | **Ethnicity** |  | ***IL36RN* genotype**^3,4^ |
| --- | --- | --- | --- | --- | --- | --- |
| 041KJO86 |  | ACH |  | European |  | p.Ser113Leu/- |
| T012695 |  | ACH |  | European |  | p.Arg102Trp/p.Ser113Leu |
| T013159 |  | ACH |  | European |  | p.Ser113Leu/p.Ser113Leu |
| T031219 |  | ACH |  | East Asian |  | c.115+6T>C/c.115+6T>C;p.Pro76Leu |
| 017KSA97 |  | GPP |  | European |  | p.Ser113Leu/p.Ser113Leu |
| 059IED50 |  | GPP |  | European |  | p.Ser113Leu/p.Ser113Leu |
| T001213 |  | GPP |  | European |  | p.Lys35Arg/p.Ser113Leu |
| T001255 |  | GPP |  | European |  | p.Ser113Leu/p.Ser113Leu |
| T001654 |  | GPP |  | European |  | p.Arg48Trp/p.Ser113Leu |
| T009357 |  | GPP |  | European |  | p.Ser113Leu/p.Ser113Leu |
| T010091 |  | GPP |  | European |  | p.Val44Met/p.Ser113Leu |
| T010111 |  | GPP |  | European |  | p.Ser113Leu/p.Ser113Leu |
| T010144 |  | GPP |  | European |  | p.Ser113Leu/- |
| T012402 |  | GPP |  | Malay |  | c.115+6T>C;p.Pro76Leu |
| T012417 |  | GPP |  | Malay |  | c.115+6T>C/c.115+6T>C |
| T012432 |  | GPP |  | Malay |  | c.115+6T>C;p.Pro76Leu |
| T012447 |  | GPP |  | Malay |  | c.115+6T>C/- |
| T012476 |  | GPP |  | East Asian |  | c.115+6T>C/c.115+6T>C |
| T012587 |  | GPP |  | European |  | p.Ser113Leu/p.Ser113Leu |
| T014402 |  | GPP |  | Malay |  | c.115+6T>C/p.Ser113Leu |
| T014413 |  | GPP |  | Malay |  | c.115+6T>C/c.115+6T>C;p.Pro76Leu |
| T014422 |  | GPP |  | East Asian |  | c.115+6T>C/- |
| T014432 |  | GPP |  | East Asian |  | c.115+6T>C;p.Pro76Leu |
| T019127 |  | GPP |  | East Asian |  | c.115+6T>C/c.115+6T>C |
| T022929 |  | GPP |  | Malay |  | c.115+6T>C/- |
| T022933 |  | GPP |  | Malay |  | c.115+6T>C/c.115+6T>C |
| T022949 |  | GPP |  | East Asian |  | c.115+6T>C/- |
| T022951 |  | GPP |  | East Asian |  | c.115+6T>C/c.115+6T>C;p.Pro76Leu |
| T025950 |  | GPP |  | East Asian |  | c.115+6T>C/c.115+6T>C;p.Pro76Leu |
| T025952 |  | GPP |  | East Asian |  | c.115+6T>C/c.115+6T>C |
| T025954 |  | GPP |  | Malay |  | c.115+6T>C/c.115+6T>C;p.Pro76Leu |
| T025956 |  | GPP |  | East Asian |  | c.115+6T>C/c.115+6T>C |
| T025960 |  | GPP |  | East Asian |  | c.115+6T>C/- |
| T025962 |  | GPP |  | Malay |  | c.115+6T>C/c.115+6T>C |
| T025964 |  | GPP |  | Malay |  | c.115+6T>C/- |
| T026274 |  | GPP |  | East Asian |  | c.115+6T>C/c.115+6T>C |
| T026275 |  | GPP |  | East Asian |  | c.115+6T>C/c.115+6T>C |
| T026278 |  | GPP |  | Malay |  | c.115+6T>C/- |
| T026281 |  | GPP |  | Malay |  | p.Ser113Leu/- |
| T028819 |  | GPP |  | European |  | p.Ser113Leu/- |
| T029999 |  | GPP |  | European |  | p.Ser113Leu/- |
| T030865 |  | GPP |  | European |  | p.Ser113Leu/- |
| T030866 |  | GPP |  | European |  | p.Ser113Leu/- |
| T030867 |  | GPP |  | East Asian |  | c.115+6T>C/c.115+6T>C |
| T031123 |  | GPP |  | Asian Other |  | c.115+6T>C/c.115+6T>C |
| T035345 |  | GPP |  | European |  | c.115+5G>A/p.Ser113Leu |
| T036388 |  | GPP |  | East Asian |  | p.Arg102Trp/- |
| T036391 |  | GPP |  | Malay |  | c.115+6T>C/c.115+6T>C |
| T036393 |  | GPP |  | Malay |  | c.115+6T>C/- |
| 025HHE49 |  | PPP |  | European |  | p.Ser113Leu/- |
| 047SJR50 |  | PPP |  | European |  | p.Pro76Leu/- |
| PUS-01 |  | PPP |  | European |  | p.Ser113Leu/- |
| PUS-23 |  | PPP |  | European |  | p.Ser113Leu/- |
| T000821 |  | PPP |  | European |  | p.Ser113Leu/p.Ser113Leu |
| T000977 |  | PPP |  | European |  | p.Ser113Leu/- |
| T000979 |  | PPP |  | European |  | p.Ser113Leu/- |
| T001209 |  | PPP |  | European |  | p.Ser113Leu/- |
| T001211 |  | PPP |  | European |  | p.Ser113Leu/- |
| T001212 |  | PPP |  | European |  | p.Ser113Leu/- |
| T002080 |  | PPP |  | European |  | p.Ser113Leu/p.Ser113Leu |
| T002787 |  | PPP |  | European |  | p.Ser113Leu/p.Ser113Leu |
| T014390 |  | ACH + GPP |  | East Asian |  | c.115+6T>C/c.115+6T>C;p.Pro76Leu |
| T022930 |  | ACH + GPP |  | East Asian |  | c.115+6T>C/c.115+6T>C |
| T026270 |  | ACH + GPP |  | East Asian |  | c.115+6T>C/c.115+6T>C |
| T001219 |  | GPP + PPP |  | European |  | p.Ser113Leu/- |
| T022940 |  | GPP + PPP |  | Malay |  | c.115+6T>C/- |

^1^Patients were classified as *IL36RN* positive if they carried at least one mutation

^2^Cases highlighted in orange have not previously been reported

^3^When a patient carries both c.115+6T>C and p.Pro76Leu, they are found on the same chromosome

^4^Based on the data generated by the Exome Aggregation Consortium (ExAc, http://exac.broadinstitute.org/), the population frequencies of recurrent *IL36RN* mutations are as follows: p.Ser113Leu: 0.006 (non-Finnish Europeans); c.115+6T>C: 0.014 (East Asians), p.Pro76Leu: 0.001(East Asians).

**Table E6.** Influence of *IL36RN* genotype on disease phenotype

|  |  |  | **Sex** | |  | **PV status** | |  | **Age of onset (yrs)** | | |  | |  |
| --- | --- | --- | --- | --- | --- | --- | --- | --- | --- | --- | --- | --- | --- | --- |
| **Diagnosis** | ***IL36RN* status** |  | **Female** | **Male** |  | **PV** | **No PV** |  | **Mean** | **SD** |  | | **Total cases** | |
| **ACH** | Positive |  | 3  (75.0%) | 1  (25.0%) |  | 3  (75.0%) | 1  (25.0%) |  | 33.0 | 27.0 |  | | **4** | |
|  | Negative |  | 11  (57.9%) | 8  (42.1%) |  | 7  (41.2%) | 10  (58.8%) |  | 60.3 | 13.1 |  | | **19** | |
| **GPP** | Positive |  | 30  (66.7%) | 15  (33.3%) |  | 18  (42.9%) | 24  (57.1%) |  | 27.3 | 21.9 |  | | **45** | |
|  | Negative |  | 98  (69.5%) | 43  (30.5%) |  | 106  (76.8%) | 32  (23.2%) |  | 32.4 | 18.5 |  | | **145** | |
| **PPP** | Positive |  | 7  (58.3%) | 5  (41.7%) |  | 4  (33.3%) | 8  (66.7%) |  | 36.6 | 20.2 |  | | **12** | |
|  | Negative |  | 168  (76.4%) | 52  (23.6%) |  | 52  (26.5%) | 144  (73.5%) |  | 43.4 | 14.9 |  | | **222** | |

Individuals with multiple diagnoses were excluded from the analysis

**Table E7:** Details of *CARD14* alleles with deleterious potential^1^

| **Sample ID**^2^ |  | **Diagnosis** |  | **Ethnicity** |  | ***CARD14* genotype** |  |
| --- | --- | --- | --- | --- | --- | --- | --- |
| T012418 |  | GPP |  | Chinese |  | p.Asp176His/- |  |
| T019112 |  | GPP |  | Chinese |  | p.Asp176His/- |  |
| T022956 |  | GPP |  | Chinese |  | p.Asp176His/- |  |
| T029079 |  | PPP |  | European |  | p.Thr591Met/- |  |
| T029361 |  | PPP |  | Unknown |  | p.Arg182Cys/- |  |
| T039935 |  | PPP |  | European |  | p.Lys78Asn/- |  |
| T039954 |  | PPP |  | European |  | p.Arg682Trp/- |  |
| T039862 |  | PPP |  | European |  | p.Arg682Trp/- |  |

^1^The table only contains variants with a CADD score >15, which is indicative of pathogenicity

^2^Cases highlighted in orange have not previously been reported

**Table E8.** Mutations observed in *AP1S3* positive patients^1^

| **Sample ID^2^** |  | **Diagnosis** |  | ***AP1S3* genotype^3^** |
| --- | --- | --- | --- | --- |
| T011528 |  | ACH |  | p.Arg33Trp/- |
| T012719 |  | ACH |  | p.Arg33Trp/- |
| T001238 |  | GPP |  | p.Arg33Trp/- |
| T010091 |  | GPP |  | p.Phe4Cys/- |
| T030865 |  | GPP |  | p.Phe4Cys/- |
| T037194 |  | GPP |  | p.Arg33Trp/- |
| 025HHE49 |  | PPP |  | p.Phe4Cys/- |
| 100IRU41 |  | PPP |  | p.Phe4Cys/- |
| PUS-06 |  | PPP |  | p.Arg33Trp/- |
| T000776 |  | PPP |  | p.Arg33Trp/- |
| T000981 |  | PPP |  | p.Arg33Trp/- |
| T000985 |  | PPP |  | p.Phe4Cys/- |
| T001004 |  | PPP |  | p.Arg33Trp/- |
| T001009 |  | PPP |  | p.Phe4Cys/- |
| T001014 |  | PPP |  | p.Phe4Cys/- |
| T002159 |  | PPP |  | p.Arg33Trp/- |
| T002206 |  | PPP |  | p.Phe4Cys/- |
| T016713 |  | PPP |  | p.Arg33Trp/- |
| T026517 |  | PPP |  | p.Arg33Trp/- |
| P020022 |  | PPP |  | p.Arg33Trp/- |
| T001882 |  | ACH + GPP |  | p.Arg33Trp/- |
| T009358 |  | ACH + GPP |  | p.Arg33Trp/- |
| T002310 |  | GPP + PPP |  | p.Phe4Cys/- |
| T009360 |  | GPP + PPP |  | p.Phe4Cys/- |

^1^All *AP1S3* positive patients were of European descent. ^2^Cases highlighted in orange have not previously been reported. ^3^The frequency of the p.Phe4Cys and p.Arg33Trp variants among non-Finnish Europeans is 0.012 and 0.011, respectively (data generated by the ExAc Consortium)

**Table E9.** Digenic mutations

| **Sample ID** |  | **Diagnosis** |  | ***IL36RN* genotype** |  | ***AP1S3* genotype** |
| --- | --- | --- | --- | --- | --- | --- |
| T010091 |  | GPP |  | p.Val44Met/p.Ser113Leu |  | p.Phe4Cys/- |
| T030865 |  | GPP |  | p.Ser113Leu/- |  | p.Phe4Cys/- |
| ERS025^1^ |  | PPP |  | p.Ser113Leu/- |  | p.Phe4Cys/- |

^1^The case highlighted in orange has not previously been reported

**Table E10.** Influence of *AP1S3* genotype on disease phenotype

|  |  |  | **Sex** | |  | **PV status** | |  | **Age of onset (yrs)** | | |  | |  |
| --- | --- | --- | --- | --- | --- | --- | --- | --- | --- | --- | --- | --- | --- | --- |
| **Diagnosis** | ***AP1S3* status** |  | **Female** | **Male** |  | **PV** | **No PV** |  | **Mean** | **SD** |  | | **Total cases** | |
| **ACH** | Positive |  | 2  (100%) | 0  (0%) |  | 2  (100%) | 0  (0.0%) |  | 65.0 | 15.6 |  | | **2** | |
|  | Negative |  | 11  (64.7%) | 6  (35.3%) |  | 6  (40.0%) | 9  (60.0%) |  | 55.6 | 16.4 |  | | **17** | |
| **GPP** | Positive |  | 4  (100%) | 0  (0%) |  | 1  (50.0%) | 1  (50.0%) |  | 39.7 | 37.8 |  | | **4** | |
|  | Negative |  | 23  (71.9%) | 9  (28.1%) |  | 10  (34.5%) | 19  (65.5%) |  | 30.2 | 26.6 |  | | **33** | |
| **PPP** | Positive |  | 13  (92.9%) | 1  (7.1%) |  | 4  (30.8 %) | 9  (69.2%) |  | 48.1 | 11.6 |  | | **14** | |
|  | Negative |  | 149  (75.3%) | 49  (24.7%) |  | 43  (23.8%) | 138  (76.2%) |  | 43.8 | 15.1 |  | | **198** | |

Individuals with multiple diagnoses were excluded from the analysis. Given that *AP1S3* mutations were only found in Europeans, the analysis was restricted to this ethnic group.
